# Regulating Maf1 Expression and Its Expanding Biological Functions

**DOI:** 10.1371/journal.pgen.1004896

**Published:** 2015-01-08

**Authors:** Robyn D. Moir, Ian M. Willis

**Affiliations:** 1 Department of Biochemistry, Albert Einstein College of Medicine, Bronx, New York, New York, United States of America; 2 Department of Systems and Computational Biology, Albert Einstein College of Medicine, Bronx, New York, New York, United States of America; Stanford University Medical Center, United States of America

Maf1 is a nutrient- and stress-sensitive global repressor of transcription by RNA polymerase (pol) III [1], [2]. Its primary function in this context is to limit the synthesis of highly abundant 5S rRNA and tRNAs in response to nutrient availability and cellular stress [3]. Thus, Maf1 ensures the efficient use of metabolic resources while balancing the need for protein synthetic components during cell growth, proliferation, differentiation, and quiescence. Less abundant products of pol III transcription (such as the spliceosomal U6 snRNA and the 7SL RNA component of signal recognition particle) are also repressed by Maf1, since its mechanism of action involves direct inhibitory interactions with proteins required for transcription by all pol III genes (i.e., the TFIIB-related initiation factors Brf1 or Brf2 and the polymerase) [4]–[7]. In the previous issue of *PLOS Genetics*, Palian et al. describe new insights into the regulation of Maf1 and its function in mammalian systems [8].

**Figure 1:**
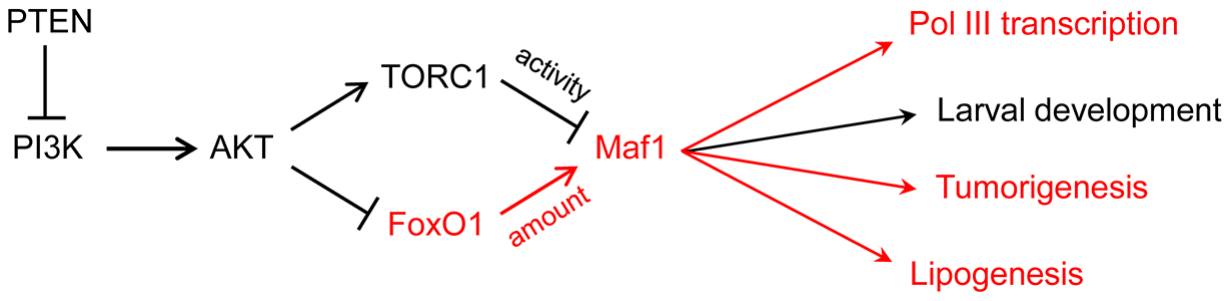
PI3K signaling via FoxO1 regulates Maf1 abundance and downstream processes. FoxO1 signaling to Maf1 and the biological processes that are sensitive to this regulation are shown in red. The larval development phenotype in *Drosophila* is primarily due to increased pol III transcription and elevated initiator tRNA^Met^. Maf1-regulation of tumorigenesis, previously thought to result from changes in pol III transcription, may also include direct effects of Maf1 on pol II transcription.

Studies on Maf1 regulation up until now have focused on posttranslational mechanisms, notably phosphorylation, which controls Maf1 localization (in yeast) and its interaction with the polymerase (in yeast and humans) [2], [3]. The new work from Palian and colleagues shows that the steady-state level of the Maf1 protein is also regulated. This is achieved through PI3K/AKT/FoxO1 signaling (Fig. 1). To reach this conclusion, tissue-specific PTEN knockout mice and a human PTEN null mutant cell line with inducible PTEN expression were used to perturb PI3K/AKT/FoxO1 signaling and show that Maf1 expression can be varied in both directions. Other manipulations of signaling through the pathway yielded consistent results. Importantly, mouse embryo fibroblasts in which the AKT substrate FoxO1 was knocked down or constitutively active showed reduced and elevated Maf1 protein levels, respectively, with corresponding reciprocal effects on the levels of precursor tRNAs (reflecting pol III transcription). Finally, the physiological relevance of the regulation was demonstrated by feeding mice a diet high in carbohydrates, which activates the pathway, and finding that Maf1 expression was decreased. One intriguing aspect of the work is that changes in PI3K/AKT/FoxO1 signaling affected Maf1 protein levels but had little influence on Maf1 mRNA. Additional studies are needed to determine how FoxO1, an insulin-sensitive DNA-binding transcription factor, alters the synthesis or stability of the Maf1 protein.

In addition to its regulation by nutrients and stress, pol III transcription is inhibited by tumor suppressors, increased by oncogenic activation and cell transformation, regulated during the cell cycle, and targeted by viruses and other pathogens [9]–[13]. The extent to which Maf1 is involved in these transcriptional changes is either not well understood or has not been examined. However, the potential for Maf1 to impact cancer-related phenotypes is suggested by several observations including (i) its control by conserved oncogenic signaling pathways, e.g., the Ras/PKA pathway as demonstrated in yeast and the TOR pathway as shown in yeast, flies and mammalian cells [3], [14]–[16]; (ii) the requirement for elevated levels of pol III transcripts for Myc-driven cell transformation and tumorigenesis [17]; (iii) the increased growth of cells with elevated levels of initiator methionine tRNA [16], [18], [19]; and (iv) the ability of Maf1 overexpression to suppress anchorage-independent growth of PTEN-deficient human glioblastoma cells [20]. Expanding on this, Palian et al., report that Maf1 levels are reduced in PTEN-negative human prostate and liver cancers compared to matched normal tissue. Moreover, they show that hepatoma cells engineered to overexpress Maf1 exhibit less anchorage-independent growth and delayed onset of tumorigenesis when the cells are injected into mice (Fig. 1). These new experiments add to the growing importance of the pol III system in cancer biology and highlight its potential as a target for cancer therapeutics. One surprising aspect of these experiments is the low level of Maf1 overexpression that was apparently needed to affect a change in function. In future studies, it will be interesting to benchmark the phenotypic changes against specific cellular quantities of Maf1.

Beyond its role in the pol III system, previous work by Johnson et al. [20] found that knockdown and overexpression of Maf1 in human cell lines affected pol II transcription of the TBP and Egr1 genes. Maf1 is not known to be a DNA-binding protein. However, its effect on transcription at the human TBP promoter is thought to be direct since it was delimited to a promoter proximal region that crosslinked to Maf1 in chromatin immunoprecipitations (ChIPs). Since this initial report, few details have emerged on the scope of Maf1 in pol II gene regulation. One recent exception is the finding that deletion of *MAF1* in yeast affects the expression of gluconeogenic genes [21]. The work from Palian et al., provides new knowledge in this regard, showing that genes encoding key lipogenic enzymes, acetyl CoA carboxylase (ACC1) and fatty acid synthase (FASN), are repressed by Maf1 (Fig. 1). As with the effect on pol III transcription, increasing or decreasing Maf1 expression had reciprocal effects on the expression of both enzymes. Moreover, these changes impacted the accumulation of lipid droplets in mammalian cell lines and triglyceride levels in mouse liver. Given the effect of Maf1 on pol III transcription and TBP expression, it is possible for Maf1 to affect the expression of additional genes by indirect mechanisms. A striking example of this capacity is the cell non-autonomous phenotype of a *Maf1* knockdown in the fat body of *Drosophila*. In this case, increased organismal growth and accelerated larval development resulted from a diffusible signal, generated in the fat body, that affected systemic signaling by insulin-like peptides synthesized in the brain [16]. Arguing against an indirect effect of Maf1 in lipogenesis in mammalian cells is the fact that the protein ChIPs to the *Fasn* promoter. Clearly, genome-wide transcriptional profiling in cells with altered Maf1 expression is necessary to better appreciate the function of this global regulator.

Altogether, the new study identifies an important mechanism of Maf1 regulation along with new Maf1-regulated protein-coding genes that impact a novel biological function. Since research on Maf1 has only just scratched the surface for a limited number of model organisms, it seems certain that additional regulatory targets and biological functions have yet to be discovered in your favorite eukaryote.
